# Supplementary material for: Chondrule formation by collisions of planetesimals containing volatiles triggered by Jupiter’s formation
Source: Sci Rep. 2025 Aug 25;15:30919. doi: 10.1038/s41598-025-12643-x (PMC12379249; doi:10.1038/s41598-025-12643-x)
Supplement: Supplementary file 1 — Supplementary Material 1 [file 41598_2025_12643_MOESM1_ESM.pdf]

Supplementary Information

Chondrule formation by collisions of planetesimals containing volatiles triggered by Jupiter's formation

Sin-iti Sirono[1*] and Diego Turrini[2]

[1]Graduate School of Earth and Environmental Sciences, Nagoya University, Nagoya, Japan.

[2]Turin Astrophysical Observatory, National Institute of Astrophysics (INAF), Pino Torinese, Italy.

Dependence of the expansion velocity of the melt layer on the volatile mass fraction

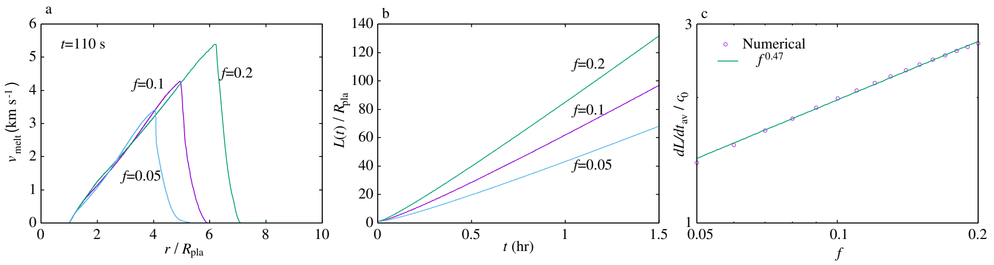

Fig. S1 Expansion of the melt layer. The planetesimal radius $R_{\rm pla}$ is 100 km and the initial melt layer thickness L_0 is 10 km. a: Velocity distribution of melt $v_{\rm melt}(r,t)$ for different volatile mass fraction f. b: Evolution of the melt layer thickness $L(t)/R_{\rm pla}$ normalized by the planetesimal radius $R_{\rm pla}$ for different volatile mass fraction f. The average expansion velocity $dL/dt_{\rm av}$ is obtained by the least-square fitting of the curve. c: the normalized average expansion velocity of the melt layer $(dL/dt_{\rm av})/c_0$ normalized by the sound velocity $c_0 = \sqrt{kT_0/m_{\rm H_2O}} \simeq 1\,{\rm km\,s^{-1}}$ as a function of the volatile mass fraction f. The average velocity was computed in the range of $0 \leq t \leq 1.5$ hr. The solid line is $dL/dt_{\rm av} \propto f^{0.47}$.

The expansion of the melt layer is driven by the expansion of the volatile material. The expansion velocity determines the cooling rate of the melt and the size of the droplets, and is therefore the key parameter in the semi-analytical solutions. The expansion velocity depends on the volatile mass fraction: when the volatile mass fraction increases so does the gas pressure gradient, while the mass of the melt droplets decreases. Fig. S1a shows the velocity distribution for different values of the volatile mass fraction f at constant time $t = 110\,\mathrm{s}$, clearly illustrating both the dependence of the peak velocity from f and how the peak velocity always exceeds the sound speed ($c_0 \simeq 1\,\mathrm{km\,s^{-1}}$). Fig. S1b displays the expansion of the melt layer for the same values of the volatile mass fraction f. The slope of the curves represents the expansion velocity and immediately shows how it is almost constant until the cooling finishes (see Fig.5b in the Main Text).

Fig. S1c shows the dependence of the expansion velocity on the volatile mass fraction f. The dependence $dL/dt \propto f^{0.47}$ resulting from the numerical simulations is in agreement with the analytical estimation of $dL/dt \propto \sqrt{f}$. The expansion velocities are $1.4c_0$, $2.0c_0$, and $2.7c_0$ for $f = 0.05$, 0.1, and 0.2, respectively. Note that the velocity is not produced by the impact itself but arises from the thermal expansion of the volatile material using the heat contained in the melt droplets.

The expansion velocity in panel c is smaller than the peaks shown in panel a. The peaks correspond to the shock wave surface. Because the shock wave travels much faster than the gas+melt mixture, the spatial density around the peak is far from the melt layer surface, where the spatial density of melt is that in the protoplanetary disk $\rho_{\mathrm{melt},\infty} = 2 \times 10^{-9}\,\mathrm{kg\,m^{-3}}$, which is much smaller than the density of the melt layer $2.7 \times 10^{-3}\,\mathrm{kg\,m^{-3}}$ at $t = 1\,\mathrm{hr}$ (Fig. 5d: the initial density is $\rho_{\mathrm{melt},0} = 2.650\,\mathrm{kg\,m^{-3}}$). The melt layer surface $L(t)$ locates at $L(t)/R_{\mathrm{pla}} = 1.5$, 1.8, and 2.2 for $f = 0.05$, 0.1, and 0.2, respectively in Fig. S1a.

The inner Solar System after Jupiter's formation

The dynamical and collisional environment responsible for the production of chondrules proves highly effective in altering the asteroid belt. At the beginning of our simulations the mass of solids contained in the orbital region of the asteroid belt between 2 and 3 au is about 2.4 $M_\oplus$. At the end of Jupiter's formation process and of the peak of chondrule production, a large fraction of this initial mass has been converted into collisional debris while planetesimals originating from beyond 3 au have been implanted into the orbital region of the asteroid belt. Specifically, in the Jovian in situ formation scenario the mass contained in the asteroid belt decreases to about 2 $M_\oplus$, of which about 0.9 $M_\oplus$ have been implanted from beyond 3 au. In the Jovian extensive migration scenario the final mass contained between 2 and 3 au drops to 0.6 $M_\oplus$, of which 0.3 $M_\oplus$ are implanted from beyond 3 au.

In the Jovian in situ formation scenario the implanted bodies originate from the now depopulated region between 3 and 5 au, while in the Jovian extensive migration scenario they originate from the region comprised between 3 and 30 au (see Fig. S2). Both scenarios are globally compatible with the observed overlapping distributions of S and C type asteroids[1] but predict the implantation of different types and abundances

of ices in the asteroid belt. The presence of ammoniated minerals on Ceres[2] and other large main belt asteroids[3] appears to favour the Jovian extensive migration scenario due to the colder origin of its implanted bodies. The current constraints on the temperature profile of the solar nebula, however, do not permit to exclude Jupiter's in situ formation as they allow for colder disks where the NH_3 snowline falls between 3 and 4 au[4].

The collisional environment responsible for the production of chondrules has also implications for the ongoing planet formation process in the inner Solar System. Specifically, as the debris and pebbles are more affected by the disk gas than the primordial planetesimals, the resulting inward transport of material can promote the growth of surviving large planetesimals and existing planetary embryos during the lifetime of the solar nebula[5], consistently with recent constraints on the accretion timescales of the terrestrial planets[6]. The resulting gravitational interactions between the growing embryos and Jupiter are known to enhance and sustain the dynamical excitation of the inner solar system[7, 8], hence the chondrule production process, beyond the 0.5 Myr caused by Jupiter alone while triggering the depletion of the asteroid belt[7, 8].

Additional processes that can sustain and extend the duration of the chondrule production, alone or in conjunction with the role of planetary embryos, are the formation of Saturn ([9–11], see Main Text) and the dynamical excitation caused by the sweeping secular resonances during the dissipation of the solar nebula after the formation of the giant planets[12]. Alternatively, the depletion of the asteroid belt and the extension of the chondrule production can be achieved by further inward migration of Jupiter as that invoked to explain the small mass of Mars[13].

Finally, these results have implications also for the interpretation of the origins of CB chondrules. CB chondrules were formed ~ 3.8 Myr after CAIs[14] and considered as the products of planetesimal collisions[15]. A detailed analysis of CB chondrules revealed that collisions with high water content ($\sim 20\,\%$) and a high cooling rate match their zoning profiles. This is consistent with our results on the f dependence of the cooling rate (Fig. 4d in the Main Text) and the dynamical implantation of volatile-rich planetesimals in the inner Solar System. Their age, however, is inconsistent with them being associated with Jupiter's formation as previously proposed[16], and indicates instead that they are the product of the formation of Saturn (see Main Text) or of the later collisional environment responsible for the depletion of the asteroid belt.

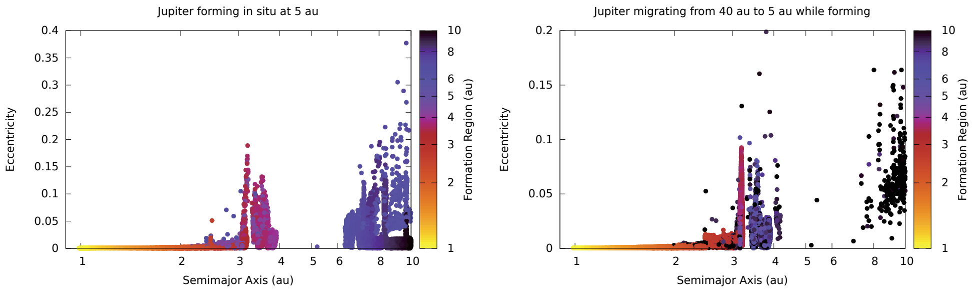

Fig. S2 The solar nebula after Jupiter's formation. The left plot shows the dynamical state of the planetesimal disk in the solar nebula in the in situ formation scenario, the right plot in the extensive migration scenario. The semimajor axes are plotted in logarithmic scales to zoom on the inner Solar System. The formation region of the planetesimals is indicated by the color scale, with the black color identifying planetesimals formed at or beyond 10 au. Planetesimals from beyond the water snowline are systematically implanted in the orbital region of the current asteroid belt between 2 and 3 au.

References

[1] DeMeo, F. E., Alexander, C. M. O., Walsh, K. J., Chapman, C. R. & Binzel, R. P. The Compositional Structure of the Asteroid Belt. in *Asteroids IV* eds. P. Michel, F. E. Demeo, and W. F. Bottke. pp.13–41 (2015). doi:10.2458/azu_uapress_9780816532131-ch002

[2] de Sanctis, M. C. *et al.* Ammoniated phyllosilicates with a likely outer Solar System origin on (1) Ceres. *Nature* **528**, 241–244 (2015). doi:10.1038/nature16172

[3] Kurokawa, H. *et al.* Distant Formation and Differentiation of Outer Main Belt Asteroids and Carbonaceous Chondrite Parent Bodies. *AGU Advances* **3**, e2021AV000568 (2022). doi:10.1029/2021AV000568

[4] Öberg, K. I. & Wordsworth, R. Jupiter's Composition Suggests its Core Assembled Exterior to the N_2 Snowline. *Astron. J.* **158**, 194 (2019). doi:10.3847/1538-3881/ab46a8

[5] Turrini, D. and 35 colleagues 2023. The GAPS programme at TNG. XLVIII. The unusual formation history of V1298 Tau. Astronomy and Astrophysics 679. doi:10.1051/0004-6361/202244752

[6] Lammer, H., Brasser, R., Johansen, A., Scherf, M. & Leitzinger, M. Formation of Venus, Earth and Mars: constrained by isotopes. *Space Science Reviews* **217**, 7 (2021). doi:10.1007/s11214-020-00778-4

[7] O'Brien, D. P., Morbidelli, A. & Bottke, W. F. The primordial excitation and clearing of the asteroid belt—Revisited. *Icarus* **191**, 434–452 (2007). doi:10.1016/j.icarus.2007.05.005

[8] Johnson, B. C., Minton, D. A., Melosh, H. J. & Zuber, M. T. Impact jetting as the origin of chondrules. *Nature* **517**, 339-341 (2015). doi:10.1038/nature14105

[9] Coradini, A., Turrini, D., Federico, C. & Magni, G. Vesta and Ceres: crossing the history of the solar system. *Space Science Reviews* **163**, 25–40 (2011). doi:10.1007/s11214-011-9792-x

[10] Turrini, D., Coradini, A. & Magni, G. Jovian early bombardment: planetesimal erosion in the inner asteroid belt.*Astrophys. J.* **750**, 8 (2012). https://doi.org/10.1088/0004-637X/750/1/8

[11] Ronnet, T., Mousis, O., Vernazza, P., Lunine, J. I. & Crida, A. Saturn's formation and early evolution at the origin of Jupiter's massive moons. *Astron. J.* **155** 224 (2018). doi:10.3847/1538-3881/aabcc7

[12] Nagasawa, M., Tanaka, H. & Ida, S. Orbital Evolution of Asteroids during Depletion of the Solar Nebula. *Astron. J.* **119**, 1480–1497 (2000). doi:10.1086/301246

[13] Walsh, K. J., Morbidelli, A., Raymond, S. N., O'Brien, D. P. & Mandell, A. M. A low mass for Mars from Jupiter's early gas-driven migration. *Nature* **475**, 206–209 (2011). doi:10.1038/nature10201

[14] Wölfer, E., Budde, G. & Kleine, T. Age and genetic relationships among CB, CH and CR chondrites. *Geochim. Cosmochim. Acta* **361**, 288–301 (2023). doi: 10.1016/j.gca.2023.10.010

[15] Fedkin, A. V., Grossman, L., Humayun, M., Simon, S. B. & Campbell, A. J. Condensates from vapor made by impacts between metal-silicate-rich bodies: comparison with metal and chondrules in CB chondrites. *Geochim. Cosmochim. Acta* **164** 236-261 (2015). doi: 10.1016/j.gca.2015.05.022

[16] Johnson B. C., Walsh K. J., Minton D. A., Krot A. N., & Levison H. F. Timing of the formation and migration of giant planets as constrained by CB chondrites. *Science Advances* **2**, e1601658 (2016). doi: 10.1126/sciadv.1601658
